# Macroevolution of sexual size dimorphism and reproduction-related phenotypic traits in lizards of the Chaco Domain

**DOI:** 10.1186/s12862-018-1299-6

**Published:** 2018-12-07

**Authors:** Guadalupe López Juri, Margarita Chiaraviglio, Gabriela Cardozo

**Affiliations:** Laboratorio de Biología del Comportamiento; Instituto de Diversidad y Ecología Animal (IDEA), CONICET-UNC and Facultad de Ciencias Exactas, Físicas y Naturales, Universidad Nacional de Córdoba, X5000JJC. Av. Vélez Sársfield 299, Córdoba, Argentina

**Keywords:** Sexual size dimorphism, Macroevolution, Intrasexual selection, Fecundity selection, Reproductive mode, Chaco Domain

## Abstract

**Background:**

Comparing sexual size dimorphism (SSD) in the light of the phylogenetic hypothesis may help to understand the phenotypic evolution associated with sexual selection (size of whole body and of reproduction-related body parts). Within a macroevolutionary framework, we evaluated the association between the evolution of SSD and the evolution of reproduction-related phenotypic traits, and whether this association has favored female fecundity, considering also variations according to reproductive modes. We focused on the lizard species that inhabit the Chaco Domain since this is a natural unit with a high diversity of species.

**Results:**

The residual SSD was related positively with the residuals of the reproduction-related phenotypic traits that estimate intrasexual selection and with the residuals of inter-limb length and, according to fecundity selection, those residuals were related positively with the residuals of clutch size in oviparous species. Lizards of the Chaco Domain present a high diversity of SSD patterns, probably related to the evolution of reproductive strategies.

**Conclusions:**

Our findings highlight that the sexual selection may have acted on the whole-body size as well as on the size of body parts related to reproduction. Male and female phenotypes evolutionarily respond to variations in SSD, and an understanding of these patterns is essential for elucidating the processes shaping sexual phenotype diversity from a macroevolutionary perspective.

## Background

Sexual size dimorphism (SSD) is generally related to way in which each sex gets the reproductive success [1, 2]. The body size associated with maximum fitness (i.e. the optimal body size) thus often differs between the sexes [3]. From a macroevolutionary perspective in terms of SSD, it is interesting to analyze the magnitude and the direction of the evolutionary dynamics of male and female phenotypes. Comparing species phenotypes, considering sexual differences, in the light of the phylogenetic hypothesis will contribute to understanding the evolutionary relationships associated with sexual selection.

Since the magnitude of sexual dimorphism is an emergent property that varies depending on the body size of the sexes, one main goal from an evolutionary perspective is to elucidate whether sexual dimorphism is also dependent on species size. According to Rensch’s rule, when males are the larger sex, SSD increases with increasing body size (hyperallometry; [4]); conversely when females are the larger sex, SSD decreases with increasing body size (hypoallometry; [5]). The body size of an animal is a key ecological and evolutionary trait for the species, evolving towards an optimum, depending on the ecological contexts and reproductive strategies [6, 7]. Therefore, the evolution of different strategies between the sexes and the consequent variations in the direction and magnitude of sexual size dimorphism may be related to species-specific body size.

Evolutionary hypotheses have been proposed for SSD changes in Squamata. Cox et al. [8] defined: 1) Intrasexual Selection Hypothesis: the strength of sexual selection diverts body size towards males, for large male size, which confers an advantage in intrasexual mate competition; and 2) Fecundity Advantage Hypothesis: the strength of sexual selection diverts body size towards females, for large female size, which confers a fecundity advantage. Evolutionary changes in clutch/litter size seem to be generally accompanied by the predicted interspecific shifts in SSD [8]. However, correlations between evolutionary changes in body size and changes in reproduction-related phenotypic traits (morphological traits involved in reproduction) that contribute to male-male competition or female fecundity have rarely been investigated empirically.

Lizards can present sexual dimorphism in the whole-body size [9] as well as in the relative size of reproduction-related body parts involved in behaviors such as mate competition, courtship and copula (e.g., head, abdomen, tail and limbs), and this can be very informative about the selective pressures imposed [10–12]. Sexual dimorphism in different morphological traits of males and females, related to reproduction, has been interpreted as a product of differential pressures between the sexes [13]. In fact, Cardozo et al. [14] suggested that different selective pressures might act on each sex, shaping the morphological traits as sexually dimorphic.

In the context of Intrasexual Selection, male-biased SSD species are expected to present a hyperallometric growth of structures used for aggressive agonistic encounters with other males [4, 8]. Increased male head size may be important in aggressive interactions, since it has been associated with bite force used during combat [15–18]. Size of hind limbs may be important, because muscle mass may help males to run at high speed [19] and consequently to dominate in combat over territories [20, 21]. However, reproduction-related phenotypic traits may be simultaneously important in intersexual interactions [22] and in intrasexual interactions [15, 23, 24], e.g., head size is an advantageous feature during mating as a signal of quality for females. Similarly, the size of the extremities may also be important for the success of copula, being involved in subjection of the female [25, 26]. The tail may be used both in courtship and/or for defense of the territory [27–29]. Consequently, we predict an association between the evolution of the sexual dimorphism of male-biased SSD species and the evolution of morphological traits such as the size of the head, hind limbs and tail.

From the perspective of Fecundity Advantage Selection, female-biased SSD species are expected to present hyperallometric growth of structures related to reproductive investment. The volume of the body can determine the physical limit to reproduction, i.e., a large body cavity allows females to store larger energy reserves or develop eggs/embryos [14, 30–32]. Consequently, a large trunk length or a greater abdominal perimeter would give the females higher fecundity [33–36]. Furthermore, Cardozo et al. [14] show that, in female lizards, the tail perimeter is positively related with body condition and consequently with stores of energy reserves to reproduction. In relation to the reproductive mode, viviparous females would require larger abdominal cavity than oviparous females because of having longer gestation period [36–38]. Viviparous females may retain the eggs within the uterus or present different degrees of placentation [39], in any case, embryos are retained to an advanced development [40, 41]. We hypothesize an association between the evolution of sexual dimorphism in female-biased SSD species and the evolution of morphological traits such as trunk length, abdomen width and tail perimeter. We also expect that this association will be different between the reproductive modes.

From a macroevolutionary perspective, several studies in lizard families show that the majority of males are larger than females, although female-biased SSD is common and occurs in nearly every family. Moreover, male-biased SSD reaches extremes in which males are an average 50% longer than females. In female-biased SSD, in contrast, females may exceed males by as much as 20%. Most families show consistent patterns of male-biased SSD, but some exhibit considerable variation with no clear directional trend in SSD [3].

In the present study, we examined how the lizard species of the Chaco Domain evolved in relationship to patterns of SSD. The Chaco Domain is a natural unit in which the inhabitant species share common patterns and processes. The domain is a homogenous unit with common features of panbiogeography, endemics and cladistic biogeography [42, 43]. This perspective is important for the present study because the processes of evolutionary dynamics that shape the diversity of phenotypes depend on the geographical aspects of the domains [44, 45]. The Chaco Domain is an important ecological-evolutionary scenario with a high diversity of species with a variety of ecological strategies [46–48] which is interesting for studying variations in the macroevolutionary patterns of sexual dimorphism in lizards.

**Fig. 1:**
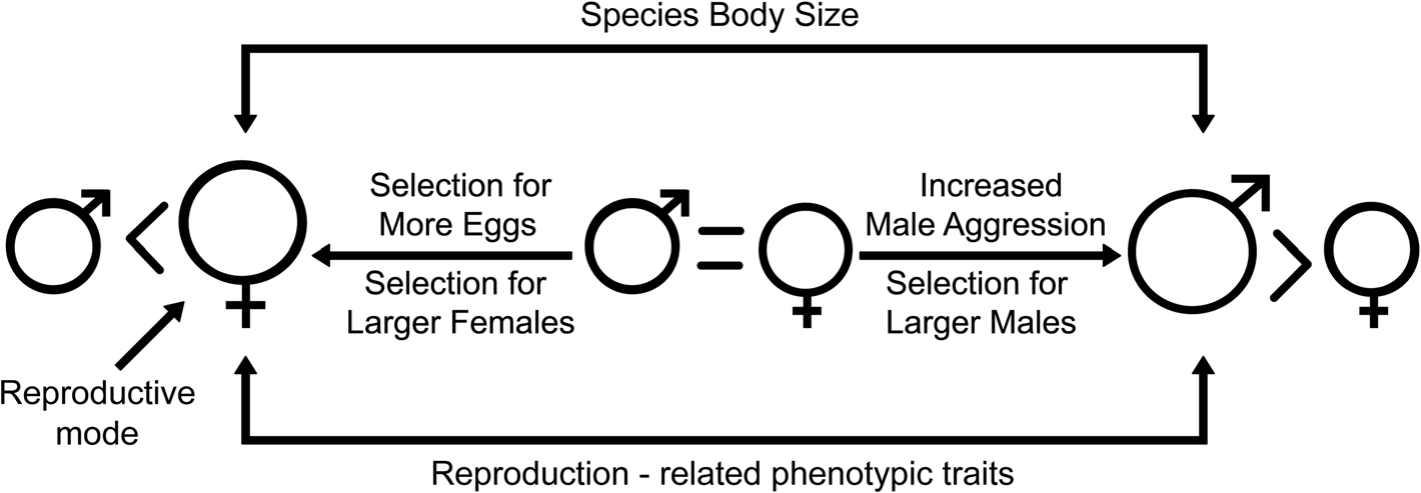
Schema of our hypothesis of evolution of sexual size dimorphism (SSD) in relation to reproduction-related phenotypic traits, reproductive mode and body size of species in the Chaco Domain (Modified from Corl et al. [82])

Within a phylogenetic comparative framework, we hypothesize that the macroevolutionary dynamics of SSD is related to evolutionary changes of species’ body size and reproduction-related phenotypic traits, which, in the case of females, may lead to variation in fecundity. Furthermore, we hypothesize that SSD patterns and the exacerbation of reproduction-related phenotypic traits differ between reproductive modes of the species (see Fig. 1). Therefore, this study aims to: i) Evaluate the magnitude and direction of SSD in lizard species of the Chaco Domain, and analyze the way in which SSD is related to body size in the species; ii) Evaluate the association between the evolution of SSD and the evolution of reproduction-related phenotypic traits; iii) Evaluate whether SSD and the exacerbation of reproduction-related phenotypic traits have favored female fecundity; iv) Evaluate the magnitude of SSD and the exacerbation of reproduction-related phenotypic traits between reproductive modes.

## Methods

### Study area

The Chaco Domain includes two disjunct areas: the first being the Caatinga region in northeastern Brazil, and the second area comprising sectors east of the Andes mountain range, southern Brazil, southeastern Bolivia, the west and center of Paraguay, much of Uruguay, and the north and center of Argentina. Our study is located in the second area, covering species belonging to the north and center of Argentina. The weather is continental, with moderate to sparse rainfall, mild winters and warm summers. The Chaco harbors an important number of species whose distributions are mostly contained within this region [49].

### Species

We used museum specimens deposited in the Instituto de Herpetología of the Fundación Miguel Lillo, Tucumán, Argentina, the Museo Argentino de Ciencias Naturales Bernardino Rivadavia (MACN), Buenos Aires, the collection of the Instituto de Bio y Geociencias del NOA (IBIGEO - CONICET), and the collection of the Instituto de Diversidad y Ecología Animal (IDEA) CONICET-UNC.

We included all the families of each infraorder of the lizard species (with the exception of Polychrotidae because it was not logistically available) and, within each family, we studied the following genera: Family Liolamidae: *Liolaemus, Phymaturus;* Family Leiosauridae: *Pristidactylus, Diplolaemus, Urostrophus;* Family Tropiduridae: *Tropidurus, Stenocercus;* Family Gymnophthalmidae: *Cercosaura, Proctoporus;* Family Teidae: *Aurivela*, *Contomastix*, *Ameiva*, *Teius;* Family Scincidae: *Notomabuya, Aspronema;* Family Phyllodactylidae: *Phyllopezus, Homonota;* 39 species were analyzed: 25 oviparous and 14 viviparous. The 577 individuals corresponded to the larger third (considering snout vent length) of the samples for each species [50], and so we are confident that only adult specimens were considered. For each species, we used an N minimum of 5 individuals for each sex. In the species presenting strong sexual dichromatism or clear dimorphic external traits, these features were used as diagnostics for sexing individuals. In those species in which external dimorphism is not recognizable, we dissected the specimens and checked gonadal structures to sex individuals.

### Traits measured

**Fig. 2:**
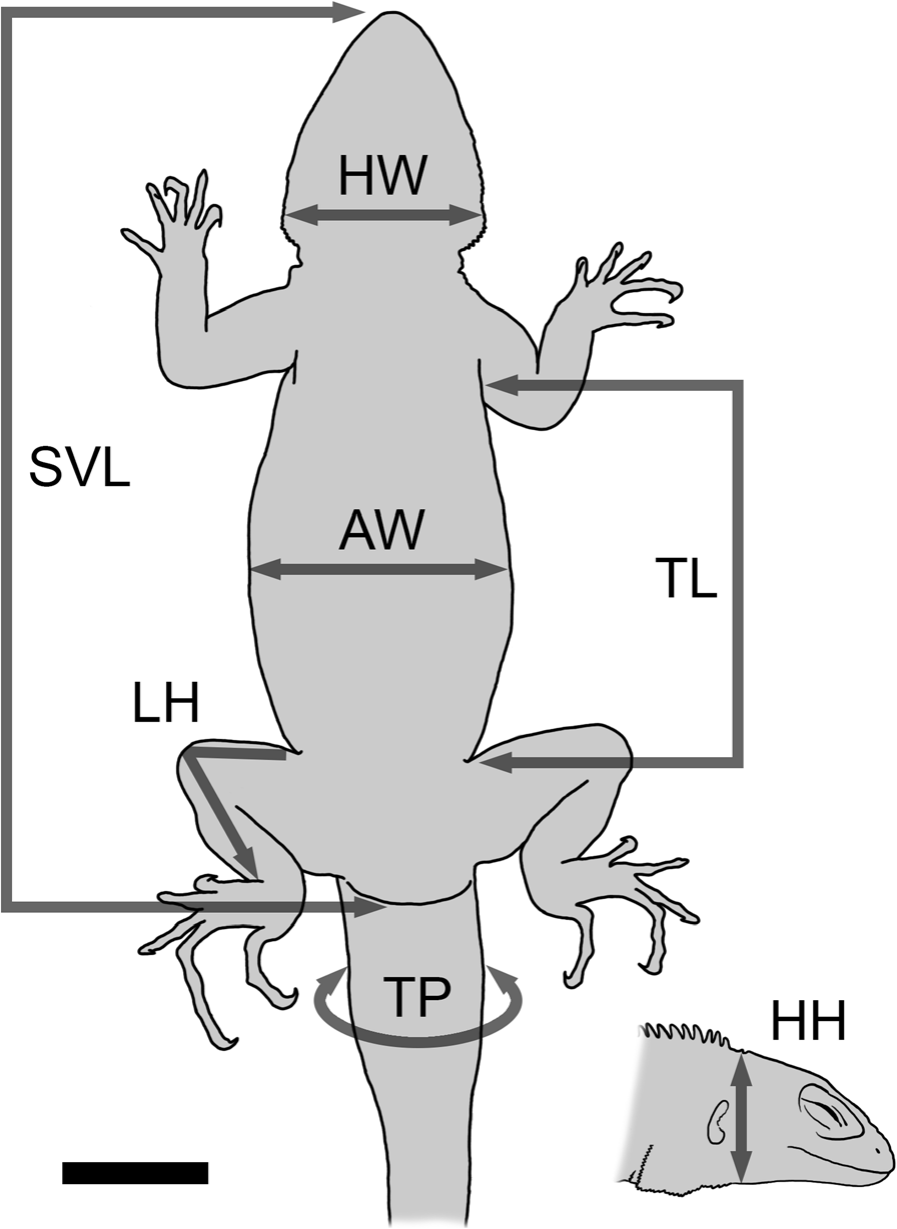
Traits of external morphology measured in each individual

Several traits of external morphology were studied in each individual: snout-vent length (SVL), head width (HW), head height (HH), trunk length (TL), abdominal width (AW), tail perimeter (TP) and total length of hindlimb (LH) (Fig. 2). The TP was measured using the centimeter-around-tail circumference and all the other traits were measured from photographs. The photographic record was analyzed with the image software Image J v.1.47 (NIH, USA). Each photo was scaled in reference to a millimeter paper and each measure of the external morphological traits mentioned was registered in triplicate.

To determine potential clutch/litter size, we dissected females corresponding to their reproductive period and quantified the vitellogenic follicles, eggs or embryos. The follicles were considered vitellogenic when they were yellow, dense and opaque inside [51, 52].

### Phylogenetic framework

**Fig. 3:**
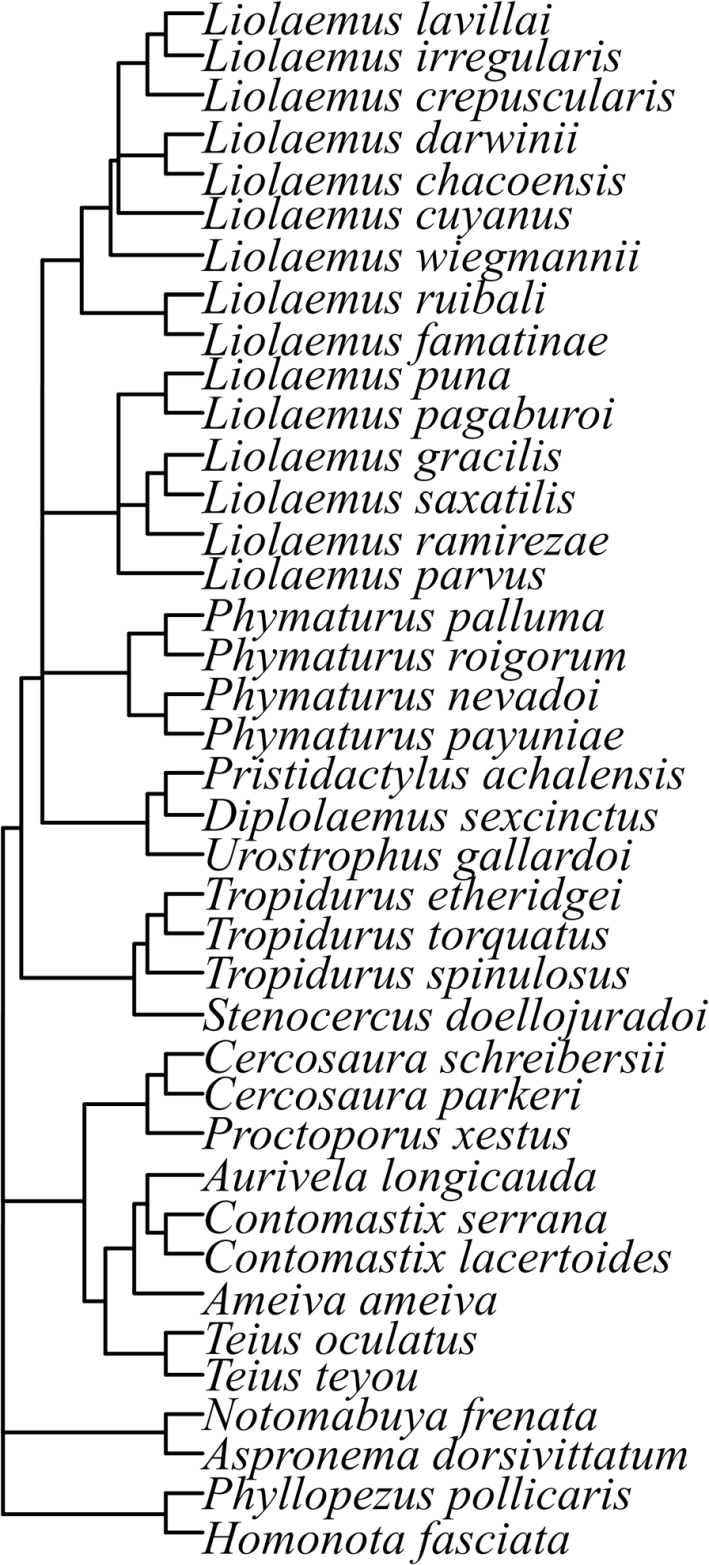
Phylogenetic hypothesis for the species of the Chaco Domain included in the study. This reconstruction was based on [53–57]

The basic phylogeny used consisted of a reduced phylogenetic tree, built respecting the topologies of the species, according to the hypothesis of the most recent relationships available [53–57]. The Felsenstein criterion was used, assuming a length of branches equal to one for the whole tree, because there was no information available on the length of branches in many of these phylogenies [58]. Figure 3 shows the reduced phylogenetic hypothesis of the species included.

### Statistical analyses

For each species, we quantified SSD with the Gibbons and Lovich index [59], SSD (Sexual Size Dimorphism) = (SVL of longest sex / SVL of shortest sex) – 1. The resulting value is then made negative if males are the larger sex and positive if females are the larger sex [3]. To evaluate the direction and magnitude of sexual dimorphism, a permutations test was performed on SSD, with *p*-value < 0.05, to determine if the sexual dimorphism index of each species was statistically significant. Those species that equaled or exceeded 15% of the SSD Index of their own family were also considered dimorphic, despite not having a significant p-value in the permutation test. We categorized the species into 3 SSD groups: male-biased SSD (when sexual dimorphism was deviated towards males); female-biased SSD (when sexual dimorphism was deviated towards females) and monomorphic (when they had no marked sexual dimorphism).

We calculated the mean values of the morphological variables per species per sex and we used Kolmogorov–Smirnov (KS) tests to ensure normality. The variables without normal distribution were logarithmically (log_10_) transformed priori to analysis. As an estimate of intrasexual selection of reproduction-related phenotypic traits, we calculated an Index sexual dimorphism to HW, HH, LH and TP_male_, as the ratio of HW in males to HW in females (IHW=HW_males_ /HW_females_) (see [17, 60, 61]), and the same for the other variables. To estimate the fecundity selection of reproduction-related phenotypic traits, we calculated an Index sexual dimorphism to TL, AW and TP_female_, as the ratio of TL in females to TL in males (ITL = TL_females_ /TL_males_), and the same for the other variables.

When examining data from phylogenetically related species, data points cannot be considered as statistically independent due to shared evolutionary history [58, 62]. So we performed the phylogenetic size-correction analysis [63] by using phylo.resid (a module of Phytools for R developed by Revell [64]), over SSD, IHW, IHH, ILH, ITP_male_, ITL, IAW, ITP_female_ and clutch/litter size. The resultant residuals from the phylogenetic size-correction were then used in the subsequent analyses.

To analyze the relationship between SSD and body size of the species, we ran Phylogenetic Generalized Least Squares (PGLS) using a model with SVLLog_10_ as predictor variable and SSD as dependent variable. To analyze the effect of sexual dimorphism on reproduction-related phenotypic traits, PGLS was run using models with residuals of IHW, IHH, ILH, ITP_male_ and of ITL, IAW, ITP_female_ as dependent variables and the residual SSD and SSD groups (males-biased SSD, females-biased SSD and monomorphic) as predictor variables.

To analyze the effect of sexual dimorphism and female reproduction-related phenotypic traits (only those significantly related to residual SSD according to the previous PGLS) on fecundity, we ran PGLS using models with residual clutch/litter size as the dependent variable and the residual SSD and SSD groups (males-biased SSD, females-biased SSD) as predictor variables. We also ran PGLS using models with residual clutch/litter size as the dependent variable and the residual ITL and SSD groups (males-biased SSD, females-biased SSD) as predictor variables. In these analyses, we eliminated the monomorphic species group because there was not enough data.

To analyze the effect of sexual dimorphism on female reproduction-related phenotypic traits (only those significantly related to residual SSD according to the previous PGLS) considering the species’ reproductive mode, we ran PGLS using models with the residual ITL as dependent variable and the residual of SSD and the reproductive mode (oviparous and viviparous) as predictor variables. Also, to analyze the effect of female reproduction-related phenotypic traits of (only those significantly related to residual SSD according to the previous PGLS) on fecundity, we ran PGLS using models with the residual clutch/litter size as dependent variable and the residual of ITL and the reproductive mode (oviparous and viviparous) as predictor variables.

We estimated Pagel’s phylogenetic signal (λ) from the residual errors simultaneously on the regression parameters of phylogenetic generalized least squares models (PGLS) analyses. Analyses were made in ‘caper’ [65] and ‘ape’ [66] packages, both developed in R [67].

## Results

### Magnitude and direction of SSD in lizard species of the Chaco Domain

**Fig. 4:**
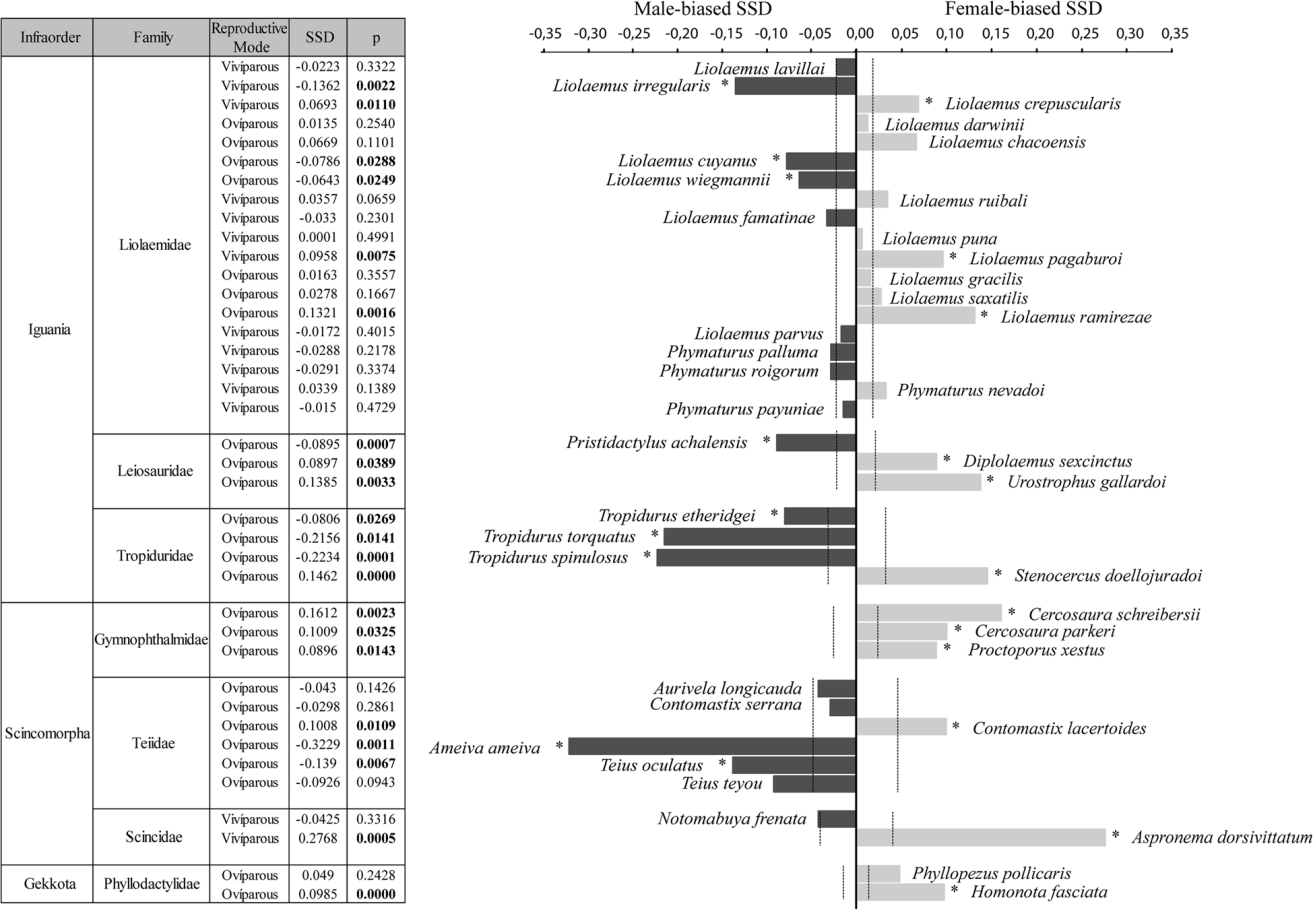
Evolutionary patterns of sexual size dimorphism in the Chaco Domain. The symbol * and the p in bold (Permutations test) indicates the species in which there is a significant difference in SVL between males and females. The dotted line (---) indicates the criterion of sexual size dimorphism, where those species that equaled or exceeded 15% of the SSD Index of their own family were also considered dimorphic, despite not having a significant *p*-value in the Permutation test

Our results showed that 41% of the species (*N* = 16) presented male-biased SSD, 41% (N = 16) female-biased SSD and 18% (*N* = 7) were monomorphic (Fig. 4). Within each family, sexual dimorphism was not consistent, since most showed both male-biased and female-biased SSD. The magnitude of the index of sexual dimorphism in species with male-biased SSD was up to − 0.33, and in species with female-biased SSD, it reached 0.30.

**Fig. 5:**
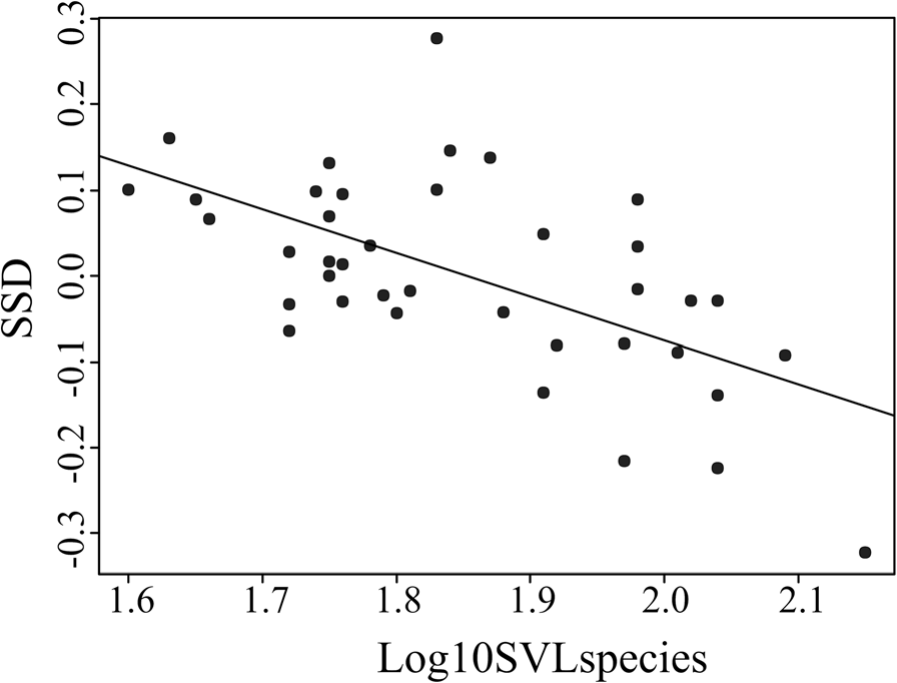
Relationship between sexual size dimorphism (SSD) and the body size of the species (Log10SVLspecies). The line represents the slope and intercept of the PGLS model regression

SSD was significantly related to the body size of the species (Adj. r^2^ = 0.35, *p* < 0.0001), being female-biased in species with small SVL, and male-biased in species with large SVL (Fig. 5).

### SSD in relation to reproduction-related phenotypic traits as estimators of intrasexual selection and fecundity selection

The residual SSD was positively related with the residuals of the reproduction-related phenotypic traits that estimate intrasexual selection in both male- and female-biased SSD species (Table 1). However, there were no differences between the female-biased SSD species and male-biased SSD species in investment in these characters. The residual SSD was positively related only with the residual ITL in female-biased SSD species (Table 1).

**Table 1:** Phylogenetic generalized least squares evaluating the effect of residual SSD on the residuals of the reproduction-related phenotypic traits in the context of intrasexual selection (IHW, IHH, ILH, ITP_male_) and in the context of fecundity selection (ITL, IAW, ITP_female_)

Model	λ	Adj. r 2	Factor Levels	Slope	SE	P
Intrasexual selection						
			SSD-female	0.998	0.058	< 0.001
Residual IHW ~	0.000	0.950	SSD-male	1.056	0.087	0.017
Residual SSD			Monomorphic	1.018	0.149	0.415
			SSD-female	1.044	0.079	< 0.001
Residual IHH ~	0.000	0.922	SSD-male	1.047	0.120	< 0.001
Residual SSD			Monomorphic	0.980	0.205	0.055
			SSD-female	0.961	0.079	< 0.001
Residual ILH ~	0.000	0.910	SSD-male	1.057	0.119	0.041
Residual SSD			Monomorphic	1.124	0.204	0.296
			SSD-female	0.962	0.064	< 0.001
Residual ITP_male_ ~	0.000	0.944	SSD-male	1.082	0.096	< 0.001
Residual SSD			Monomorphic	0.913	0.165	0.044
Fecundity selection						
			SSD-female	0.934	0.057	< 0.001
Residual ITL ~	0.234	0.938	SSD-male	0.929	0.085	0.2436
Residual SSD			Monomorphic	0.881	0.138	0.4
			SSD-female	0.204	0.307	0.51
Residual IAW ~	1	0.139	SSD-male	−0.439	0.395	0.731
Residual SSD			Monomorphic	1.653	1.507	0.765
			SSD-female	−0.02	0.346	0.9536
Residual ITP_female_ ~	0.933	0.195	SSD-male	−0.581	0.444	0.7686
Residual SSD			Monomorphic	1.729	1.679	0.9728

*λ* phylogenetic signal (Pagel’s), *r*^*2*^ correlation coefficient, *SE* standard error

### Relation between SSD, estimators of fecundity selection and clutch/litter size

The residual SSD was positively related with the residual clutch/litter size in female-biased SSD species (Table 2). Considering that the only estimator of fecundity related to residual SSD was ITL, we tested the effect of ITL on clutch/litter size. The residual ITL was positively related with residual clutch/litter size in female-biased SSD species (Table 3).

**Table 2:** Phylogenetic generalized least squares evaluating the effect of residual SSD on fecundity in SSD groups

Model	λ	Adj. r ^2^	Factor Levels	Slope	SE	P
Residual Clutch/litter size ~	1.000	0.862	SSD-female	1.077	0.441	0.037
Residual SSD			SSD-male	0.920	0.451	0.149

*λ* phylogenetic signal (Pagel’s), *r*^*2*^ correlation coefficient, *SE* standard error

**Table 3:** Phylogenetic generalized least squares evaluating the effect of residual ITL on fecundity in SSD groups

Model	λ	Adj. r ^2^	Factor Levels	Slope	SE	P
Residual Clutch/litter size ~	1.000	0.907	SSD-female	1.394	0.390	0.006
Residual ITL			SSD-male	1.010	0.412	0.078

*λ* phylogenetic signal (Pagel’s), *r*^*2*^ correlation coefficient, *SE* standard error

### SSD in relation to reproductive modes

The frequency of female-biased SSD, male-biased SSD and monomorphic species was similar between viviparous and oviparous species (Oviparous: SSD-female: 0.48, SSD-male: 0.36, monomorphic: 0.16; Viviparous: SSD-female: 0.36, SSD-male: 0.43, monomorphic: 0.21; χ^2 =^ 0.57, *p* = 0.7512).

There was no significant difference in SSD between the reproductive modes (Oviparous: Mean SSD ± SD = 0.01 ± 0.13, Viviparous: Mean SSD ± SD = 0.01 ± 0.09, *T* = − 0.49; *p* = 0.6239). However, in oviparous species SSD ranged from 0.15 to − 0.30, while in viviparous species it varied between 0.30 and − 0.15.

As mentioned above, considering that the sole estimator of fecundity related to residual SSD was ITL, we tested the relationship between residual SSD and residual ITL considering reproductive modes. The residual SSD was positively related with the residual ITL in oviparous but not in viviparous species (Table 4). The residual ITL was positively related with residual clutch/litter size in oviparous but not in viviparous species (Table 5).

**Table 4:** Phylogenetic generalized least squares evaluating the effect of residual SSD on residual ITL considering reproductive mode

Model	λ	Adj. r 2	Factor Levels	Slope	SE	P
Residual ITL ~	0.000	0.946	Oviparous	0.972	0.045	< 0.001
Residual SSD			Viviparous	0.869	0.079	0.313

*λ* phylogenetic signal (Pagel’s), *r2* correlation coefficient, *SE* standard error

**Table 5:** Phylogenetic generalized least squares evaluating the effect of residuals ITL on fecundity considering to reproductive mode

Model	λ	Adj. r ^2^	Factor Levels	Slope	SE	P
Residual Clutch/litter size ~	1.000	0.765	Oviparous	1.599	0.275	< 0.001
Residual ITL			Viviparous	0.926	0.458	0.679

*λ* phylogenetic signal (Pagel’s), *r*^*2*^ correlation coefficient, *SE* standard error

## Discussion

Lizards inhabiting the Chaco Domain present a great diversity in macroevolutionary patterns related to variations in sexual dimorphism, with a continuum from high male-biased SSD species to high female-biased SSD species. It is also remarkable that there is a similar proportion of species with male-biased SSD and female-biased SSD. The species with male-biased SSD and female-biased SSD have a similar magnitude of the SSD and there are even species with no marked sexual dimorphism. In contrast, in many lizard families worldwide the most common pattern is that the species frequency and intensity of SSD is mainly male-biased [3, 68]. Macroevolutionary interspecific comparisons of SSD help to understand the diversity of evolutionary patterns of sexual dimorphism across taxa. The high diversity of sexual dimorphism found in the Chaco Domain reveals it as a unit rich in evolutionary dynamics that shape the diversity of phenotypes.

The SSD pattern in the Chaco Domain supports the macroevolutionary pattern commonly named as Rensch’s rule [4, 69]. The body size of the species was related to their SSD: in species of small body size, SSD was commonly female-biased, while in large body size species, SSD was commonly male-biased. While much is known about these evolutionary patterns, it is still a challenge to elucidate the way in which entire phenotypes vary in accordance with SSD variations. Some of our results may help to explain whether body size in SSD-male species is related to increases in body parts involved in male competition or mate acquisition, and if body size in SSD-female species is related to increases in body parts that may be involved in fecundity.

Our results support intrasexual selection based on relationships between residual SSD and residuals of some reproduction-related phenotypic traits of males. The species that invested more in SSD, invested more in the exacerbation of these phenotypic traits, indicating that SSD evolves together with specific body parts, such as the head, limbs or tail. In the case of male-biased SSD lizards male body size is very important since it correlates with measures of reproductive success [70, 71]. Moreover, male body size is correlated with the size of specific body parts. For example, the allometry of head size may be favored by an advantage in male-male combats or a greater likelihood to be chosen by females [15, 21, 72–74].

Lizards in which a female-biased SSD prevails may reveal allometry of male reproduction-related phenotypic traits as a product of intersexual interaction. In female-biased SSD species, the more the females of the species invest in body size, the more the males of the same species invest in the exacerbation of reproduction-related phenotypic traits. In the case of head size, this may be related to a large head providing males with increased bite force, and therefore during intersexual interactions (i.e., courtship, copulation), males may benefit from increased bite performance, because they will be able to grasp larger females to copulate [15, 18].

In relation to fecundity selection, in the Chaco Domain the macroevolutionary morphological responses are not allometric in all the reproduction-related phenotypic traits but evolutionary responses may also be diverse in the different traits, for instance, being more accented in some traits, such as ITL, which is directly related to fecundity. We found no support for the exacerbation of abdominal width and tail perimeter associated with SSD, indicating that evolutionary changes in SSD in female body size may not be associated with evolutionary changes in abdominal capacity or with energy reserves related to tail size. However, we found support for the fecundity selection in that, in female-biased SSD species, the more the females invest in body size; the more they also invest in the exacerbation of ITL. When females are the larger sex, this is most commonly explained by fecundity selection acting to increase abdomen length [8].

Our macroevolutionary comparison across taxa also suggests that the selection of fecundity has had a major role in the evolution of female body shape by enlarging the abdomen and thus favoring clutch/litter size. Our results (Table 3) strongly indicate that the evolution of a large abdomen (ITL) allows females to accommodate more offspring. The abdomen limits physically the maternal allocation in the offspring; therefore abdomen size is under direct selection pressure [2, 8, 69, 75]. Actually, in lizards, when the abdomen is experimentally reduced, also clutch size decreases [76], this is because the strong correlation between the abdomen size and the clutch size [15, 70, 77, 78]. Our results complement those of Cox et al. [8] who demonstrated that SSD is positively correlated with clutch/litter size, but we found that, from a macroevolutionary perspective, ITL is a major trait involved in enabling increased clutch/litter size, similar to the findings of [36].

With respect to reproductive mode, we showed that the outcomes of evolutionary processes related to changes in sexual dimorphism differed according to reproductive modes; i.e., only oviparous species there is a relationship between residual SSD and residual ITL. Moreover, these parameter and clutch/litter sizes were interrelated, only oviparous species. This may indicate the importance of abdominal length for storing many eggs, although the development of those eggs would require less abdominal space in oviparous than in viviparous species. These results do not match our prediction. It would be expected that females of viviparous species would be larger than those of oviparous species, generating more female-biased patterns of SSD, but we found only a minimal tendency to divert towards female-biased SSD. However, viviparous species may be affected by fecundity selection through another reproductive parameter which we did not consider in this work (e.g., relative clutch/litter mass of the eggs, clutch/litter frequency, progeny size; see [79, 80]). As stated by Kupfer et al. [81] reproductive traits are likely to change in an evolutionary scenario of reproductive modes, in fact they evaluated several traits related to female investment and female size and found that variables such as hatchling size, egg volume and offspring number were influenced by the evolution of reproductive modes.

Integrated analyses across biological and evolutionary scales will serve to solidify the synthesis of evolutionary biology and shed new light on SSD patterns from a macroevolutionary perspective. Estimation of the phylogenetic signal (λ) is very valuable for elucidating the imprint of phylogeny on the body shape of relative species. In most associations between reproduction-related phenotypic traits (such as IHW, IHH, ILH, ITP_male_ and ITL) and SSD, we found a low phylogenetic signal, which may indicate that, beyond phylogeny, the evolutionary dynamic influences both SSD and reproduction-related phenotypic traits. In contrast, the high phylogenetic signal observed in other models where reproduction-related phenotypic traits (such as IAW, ITP_female_) were not associated with SSD suggests that evolutionary processes related to sexual dimorphism may influence body size differently from other phenotypic traits. Reproduction-related phenotypic traits may be under different selection pressures resulting in a diversity of patterns of sexual dimorphism of the body parts.

## Conclusions

Our study on SSD patterns of lizards of the Chaco Domain from a macroevolutionary perspective included a large number of diverse genera and families, and demonstrated the high diversity of SSD patterns, which might be related to a great diversity of reproductive strategies of the lizards inhabiting in this region. The sexual selection may have acted on whole-body size as well as on the size of body parts related to reproduction. Male and female phenotypes evolutionarily respond to variations in SSD, and an understanding of these patterns is essential for elucidating the processes shaping sexual phenotype diversity from a macroevolutionary perspective.

## Abbreviations

AW: Abdominal width
HH: Head height
HW: Head width
LH: Total length of hindlimb
PGLS: Phylogenetic Generalized Least Squares
SSD: Sexual size dimorphism
SVL: Snout-vent length
TL: Trunk length
TP_female_: Female tail perimeter
TP_male_: Male tail perimeter
